# General practitioners' knowledge and practice of complementary/alternative medicine and its relationship with life-styles: a population-based survey in Italy

**DOI:** 10.1186/1471-2296-8-30

**Published:** 2007-05-15

**Authors:** Massimo Giannelli, Marina Cuttini, Monica Da Frè, Eva Buiatti

**Affiliations:** 1 Unit of Epidemiology and Biostatistics, Department of Physical Therapy, Policlinico Italia, piazza del Campidano 6, Rome 00162, Italy; 2 Unit of Epidemiology, Bambino Gesù Children's Hospital, piazza S. Onofrio 4, Rome 00165, Italy; 3 Observatory of Epidemiology, Regional Health Agency of Tuscany, viale Milton 7, Florence 50129, Italy

## Abstract

**Background:**

The growing popularity of CAM among the public is coupled with an ongoing debate on its effectiveness, safety, and its implications on the reimbursement system. This issue is critically important for GPs, who have a "gatekeeping" role with respect to health care expenditure. GPs must be aware of medications' uses, limitations and possible adverse effects. Our objective was to explore GPs' knowledge of CAM and patterns of recommendation and practice, as well as the relationship between such patterns and GPs' life-styles.

**Methods:**

A cross-sectional study was conducted in Tuscany, a region of central Italy. One hundred percent female GPs (498) and a 60% random sample of male GPs (1310) practising in the region were contacted through a self-administered postal questionnaire followed by a postal reminder and telephone interview.

**Results:**

Overall response rate was 82.1%. Most respondents (58%) recommended CAM but a far smaller fraction (13%) practised it; yet 36% of CAM practitioners had no certificated training. Being female, younger age, practising in larger communities, having had some training in CAM as well as following a vegetarian or macrobiotic diet and doing physical activity were independent predictors of CAM recommendation and practice. However, 42% of GPs did not recommend CAM to patients mostly because of the insufficient evidence of its effectiveness.

**Conclusion:**

CAM knowledge among GPs is not as widespread as the public demand seems to require, and the scarce evidence of CAM effectiveness hinders its professional use among a considerable number of GPs. Sound research on CAM effectiveness is needed to guide physicians' behaviour, to safeguard patients' safety, and to assist policy-makers in planning regulations for CAM usage.

## Background

Complementary and alternative medicine (CAM) includes a variety of diagnostic and therapeutic practices whose underlying theory or explanatory mechanisms do not conform to current medical thinking [1]. In its various forms, CAM is enjoying a growing popularity among the public [2,3]. Dissatisfaction with mainstream modern medicine particularly with regards to patient-physician relationship, concerns about the side effects of chemical drugs, and personal beliefs favouring a more holistic orientation to health care are often quoted as possible explanations [3-5]. Estimates of CAM use in Western countries range from about one-third to half of the general population [6,7]. In Italy the proportion has almost doubled during the last decade [8], although it still remains far below the estimates reported in many European countries and the US. The analysis of data collected in 1999–2000 among the general population by the Italian National Institute for Statistics showed that in Tuscany 13.6% of adults had made use of CAM in the previous year [9]; yet the local Government is providing cost reimbursement for some CAM treatments under the National Health System for certain select conditions.

The use of CAM remains controversial [6]. Most of the debate focuses on its safety and effectiveness. Proper scientific evidence is lacking for most forms of CAM, partly because little methodologically rigorous evaluation has been carried out [6,10]. The controversy also concerns the costs and reimbursement system [7]. So far, in most countries people have been paying out of pocket for these therapies, and providers' fees and national total expenditures appear to be considerable [6,7]. There is a trend towards an increasing insurance coverage to include CAM treatments, in some cases covered by public money [7]. These issues are relevant for general practitioners (GPs), whose "gatekeeping" role with respect to health care expenditure is critically important. Faced with the increasing demand for CAM by their patients, GPs have to be prepared to discuss its uses and limitations, as well as its possible adverse effects [11]. They have to watch for signs of non-compliance with prescribed conventional treatments that may be associated with the use of CAM, as this is often not explicitly reported by patients [12]. Sometimes GPs are known to practise CAM themselves, particularly in certain countries [4,13].

Several studies have described GPs' CAM views [14] and practices [15]; however, most of them have been hampered by methodological problems such as small sample size, convenience sampling, or unsatisfactory response rates [16].

This paper reports the results of a large population-based survey carried out in Tuscany with the aim of exploring GPs' knowledge of CAM and patterns of recommendation and practice, as well as the relationship between these patterns and the personal and professional characteristics of the physicians.

## Methods

### Study population

**Figure 1:**
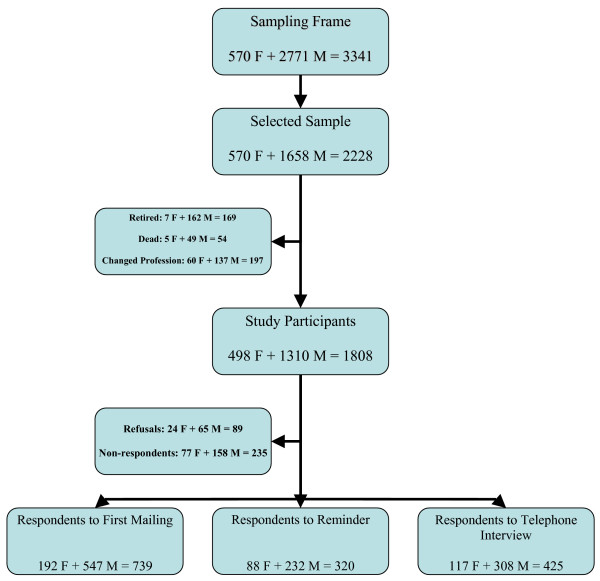
Flow-chart representing the selection process and participation status of GPs.

The survey was carried out in Tuscany, a region of central Italy with about 3.5 million inhabitants. The comprehensive sampling frame of GPs operating in the region at the time of the survey, provided by the national federation of family doctors (FIMMG), included 570 female and 2771 male doctors. A sample of 2200 subjects was needed to detect a real frequency of CAM practice of 25% ± 2% with a confidence level of 95%, allowing for 18% refusals, deaths, retirement and change of profession among GPs (percentages obtained from pilot study). As female doctors were smaller in number, it was decided to recruit all of them in order to detect possible gender differences at the analysis stage; in addition, 60% of male doctors were selected by simple random sampling without replacement. Overall 2228 subjects were contacted; GPs who had retired (169), died (54), or changed profession (197) were excluded from the study, and as a result the study population consisted of 1808 GPs (498 females and 1310 males) (figure 1).

### Questionnaire and data collection

Data were collected through a structured, self-administered postal questionnaire. The instrument was developed on the basis of a literature review and the work of a focus group of local complementary medicine therapists. It aimed at recording information on the frequency and pattern of recommendation of CAM to patients as well as GPs' personal activity as CAM practitioners. A comprehensive list of locally popular forms of CAM was provided by the focus group. The list included the following eleven types which were investigated: acupuncture, phytotherapy (also referred to as herbal medicine), homeopathy, manipulative therapies (including chiropractic and osteopathy), moxibustion, Bach's flower therapy, Shiatsu, plantar reflexology, Ayurveda, mesotherapy (technique where medication is injected into the mesoderm) using unconventional medications, and pranotherapy (energy healing based on the laying-on of hands). Respondents were given the opportunity to report additional CAM therapies practised or recommended. GPs' demographics (age and sex), lifestyle behaviours (cigarette smoking; physical activity in the past 12 months, defined as walking for at least 1000 meters and/or practice of any sport; current type of diet), and professional characteristics (years of graduation from medical school; type of postgraduate specialisation, if any; number of inhabitants in the area of practice; any certificated CAM training, completed or in progress, with specification of type and duration; for those without CAM training, interest in starting CAM training) were also recorded.

The questionnaire was piloted on 200 GPs randomly selected from the original sampling frame and no substantial modifications to the instrument were made as a result; thus, the data obtained were included in the final analysis. Data collection was carried out from February to July 2003. The questionnaire was mailed to the selected GPs together with an addressed, stamped envelope for reply, and a postal reminder followed 45 days afterwards. Non-respondents (749) were contacted by a trained interviewer for telephone administration of the questionnaire. No ethics committee approval was needed for this study.

### Statistical analysis

Statistical analysis was performed using STATA software, version 8.0 [17]. Weights were used to take into account the different sampling fractions applied to males and females; thus, unless otherwise specified, results are presented as weighed estimates with 95% confidence intervals. Chi-square test was used to check for associations in the univariate analysis. Multiple logistic regression was carried out to identify factors independently associated with two main outcome variables, namely: 1. CAM recommendation to patients (often or sometimes versus never); 2. activity as CAM practitioner (current vs never or in the past only). Factors considered for inclusion in the model as predictors were respondents' age and gender, lifestyle behaviours (currently smoking; vegetarian or macrobiotic diet; physical activity during the last year), and professional characteristics (postgraduate specialisation; any certificated CAM training, completed or in progress; number of inhabitants in the area of medical practice). The inclusion of variables in the final models was based on statistical association with the outcome of interest (p < 0.05) or evidence of confounding.

## Results

Overall, 1484 completed questionnaires were collected, corresponding to a response rate of 82% (83% for men and 80% for women); non-respondents were 13.0% and explicit refusals 4.9% (figure 1). Participants were mainly middle-aged with an overall median of 50 years, and a 25th and 75th centile of 47 and 53 years respectively (data not shown). Women were younger than men (mean age 48.2 and 52.1 years respectively, p < 0.001). Additional personal and professional characteristics of participants are shown in Table 1.

**Table 1:** Age, lifestyle behaviours and professional characteristics of participants by gender*

	**Males (N = 1087)**		**Females (N = 397)**		**Total (N = 1484)**	
	**N**	**%**	**N**	**%**	**N**	**%**

**Age and lifestyle behaviours**						
Age						
< 54	743	69.4	368	93.9	1111	76.0
≥ 54	327	30.6	24	6.1	351	24.0
Current smoking						
no	754	78.4	277	74.7	1031	77.3
yes	208	21.6	94	25.3	302	22.7
Physical activity in the past year						
no	108	11.4	65	17.9	173	13.2
yes	843	88.6	299	82.1	1142	86.8
Vegetarian/macrobiotic diet						
no	946	98.1	359	97.3	1305	97.9
yes	18	1.9	10	2.7	28	2.1
**Professional characteristics**						
Post-graduate specialisation						
no	410	37.9	112	28.3	522	35.4
yes	671	62.1	283	71.6	954	64.6
Certificated training in CAM						
no	957	89.1	336	85.1	1293	88.0
yes	117	10.9	59	14.9	176	12.0
Interest in CAM training°						
no	571	60.0	138	41.7	709	55.3
yes	252	26.5	140	42.3	392	30.5
don't know	129	13.5	53	16.0	182	14.2
No. of inhabitants in area of practice						
≤ 10.000	234	21.6	96	24.2	330	22.3
10.001 – 50.000	371	34.3	144	36.4	515	34.8
> 50.000	477	44.1	156	39.4	633	42.8

* Percentages are unweighed estimates

° Question addressed to GPs who reported no certificated CAM training

### CAM recommendation and practice

The majority of GPs reported recommending CAM to their patients sometimes (53.6%) or often (4.3%) (table 2). The most frequently recommended CAM treatment was acupuncture (69.2% GPs), followed by manipulative therapy (47.9%) and homeopathy (38.1%). Among physicians reporting never recommending CAM to patients, about two thirds were not convinced of its effectiveness (lack of evidence 47.6%; believing it is useless 19.1%), while approximately one third felt they had not enough knowledge to be able to recommend it (table 2).

**Table 2:** CAM recommendation and practice by GPs in Tuscany

	**Total**		
	**N**	**%**	**95% CI**

**Recommendation**			
Frequency of CAM recommendation to patients			
never	605	42.1	40.5–43.6
sometimes	803	53.6	52.0–55.1
often	68	4.3	3.7–4.8
Type of CAM recommended * +			
Acupuncture	599	69.2	67.3–71.0
Manipulative therapy	421	47.9	45.8–49.8
Homeopathy	343	38.1	36.1–39.9
Phytotherapy	214	23.4	21.8–25.0
Mesotherapy (unconventional medications)	78	9.1	7.9–10.2
other	175	19.6	18.0–21.1
Reasons for never recommending CAM to patients ° +			
lack of evidence of its effectiveness	275	47.6	45.1–50.0
not enough knowledge of it	211	33.4	31.1–35.6
fear it may be dangerous if used as replacement of conventional medicine	121	20.3	18.4–22.4
believing it is useless	113	19.1	17.2–21.1
fear of potential side effects	23	3.9	3.0–4.9
no chance	15	2.4	1.7–3.3
**Practice**			
Frequency of CAM practice			
never	1231	84.8	83.6–85.8
in the past only	31	2.2	1.8–2.8
currently	197	12.9	11.9–13.9
Patterns of practice among current CAM practitioners			
occasional	122	62.5	58.3–66.4
regular, as additional activity	73	36.8	32.8–40.9
regular, as main activity	2	0.7	0.7–0.7
Type of CAM practised by current CAM practitioners +			
Homeopathy	91	42.7	38.8–46.7
Phytotherapy	85	41.3	37.3–45.3
Mesotherapy (with unconventional medications)	57	30.6	26.8–34.7
Acupuncture	42	23.0	19.4–26.8
Manipulative therapy	24	13.0	10.3–16.3
other	64	32.2	28.4–36.1

+ More than one option was possible.

* Among physicians reporting to recommend CAM often or sometimes

° Among physicians who never recommend CAM to their patients

Two hundred twenty-eight physicians reported having practised some form of CAM: 2.2% had practised CAM in the past only and 12.9% were current CAM practitioners (table 2). Current practice was mostly occasional (62.5% of GPs currently practising it). The types of CAM most often practised were homeopathy (42.7% of current practitioners), phytotherapy (41.3%) and mesotherapy with unconventional medications (30.6%).

Acupuncture, manipulative therapy and mesotherapy were most frequently used by GPs to treat pain syndromes, whereas homeopathy and phytotherapy were mostly used for chronic illnesses and psychological conditions (table 3).

**Table 3:** Indications to treatment by type of CAM among Tuscan GPs currently practising it

	**Homeopathy** ** (N = 91)**			**Phytoterapy** ** (N = 85)**			**Mesotherapy** ** (N = 57)**			**Acupuncture** ** (N = 42)**			**Manipulative therapy** ** (N = 24)**		
	**n**	**%**	**95% CI**	**n**	**%**	**95% CI**	**n**	**%**	**95% CI**	**n**	**%**	**95% CI**	**n**	**%**	**95% CI**

acute illness	57	64.5	59.1–69.6	40	48.7	42.6–54.8	25	44.4	36.6–52.4	23	54.1	44.7–63.2	12	52.7	40.5–64.6
chronic illness	67	74.5	69.4–79.0	59	69.6	63.7–74.9	28	47.9	40.0–55.8	27	63.4	53.8–71.9	9	37.3	26.2–49.7
psychological condition	55	60.6	54.9–66.1	55	63.9	57.7–69.6	3	5.8	2.8–11.4	19	45.9	36.7–55.2	3	13.6	6.8–25.3
pain syndromes	51	57.9	52.2–63.3	34	40.1	34.2–46.2	43	75.3	67.7–81.5	41	97.4	91.4–99.2	22	90.9	79.9–96.1
quality of life improvement	51	56.2	50.4–61.8	48	57.3	51.2–63.2	11	17.4	12.5–23.6	10	23.7	16.6–32.5	4	18.2	10.1–30.5

**Figure 2:**
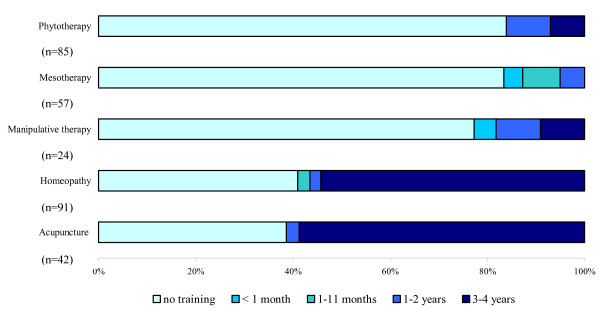
CAM training among GPs currently practising it by type of CAM.

Among the 176 physicians who reported to have training in CAM, the majority (71.9%) were also practising it at the time of the survey; however, among the 197 CAM practitioners 35.9% reported no certificated training (data not shown). Figure 2 shows CAM training and its duration among current CAM practitioners. Specific training was reported by about 60% of respondents practising acupuncture and homeopathy, 22.7% of those practising manipulative therapies, and less than 20% of those involved in mesotherapy and phytotherapy. Duration of training followed a similar pattern, with educational periods of three years or more reported by approximately 90% GPs trained in acupuncture and homeopathy, 50.0% trained in manipulative therapy and 33.3% in phytotherapy.

### Predictors of patterns of CAM recommendation and practice: multivariate analysis

Table 4 shows factors associated with GPs' recommendation of CAM to patients (often or sometimes vs never) and CAM practice (current vs never or in the past only). Training in CAM was the strongest predictor for both recommendation (OR 7.4, 95% CI 5.3–10.1) and practice (OR 47.8, 95% CI 36.5–62.4). The number of inhabitants in the area of practice was also relevant, with physicians working in larger cities (> 50000 inhabitants) being more likely to recommend and practise CAM compared to those working in medium or small size communities. Among personal characteristics, younger age (< 54) and being female were associated with an increased probability of both CAM recommendation and practice. Physicians following a vegetarian or macrobiotic diet were twice as likely to recommend and practice CAM; a similar association was found between physical activity and CAM recommendation. No association between cigarette smoking and outcome variables was found.

**Table 4:** Factors related to GPs' recommendation and practice of CAM: results from multivariate analysis

**Characteristics**	CAM recommendation (*)					**CAM practice (°)**				
	
	GPs		multivariate logistic model			GPs		multivariate logistic model		
	no.	(%)	OR^+^	95% CI	p value	no.	(%)	OR^+^	95% CI	p value

**Age**										
< 54	675	60.0	1.0		<0.001	148	13.0	1.0		0.016
≥ 54	187	52.7	0.7	0.5–0.8		46	12.8	0.7	0.5–0.9	
**Sex**										
male	602	55.7	1.0		<0.001	127	11.9	1.0		0.002
female	269	68.1	1.7	1.5–1.8		70	17.9	1.3	1.1–1.5	
**Veget./macrobiotic diet**										
no	759	57.2	1.0		0.021	174	13.0	1.0		0.012
yes	23	79.2	2.1	1.1–3.8		10	36.8	2.3	1.2–4.5	
**Physical activity**										
no	86	49.8	1.0		<0.001	25	14.6	1.0		0.707
yes	685	58.7	1.7	1.3–2.0		157	13.4	0.9	0.6–1.3	
**Certificated CAM training**										
no	709	54.1	1.0		<0.001	71	5.3	1.0		<0.001
yes	155	88.1	7.4	5.3–10.1		126	71.9	47.6	36.3–62.3	
**No. inhabitants in the area of medical practice**										
≤ 10.000	176	52.5	1.0		0.002	40	12.6	1.0		0.020
10.001 – 50.000	304	58.2	1.2	0.9–1.4		74	13.8	1.5	1.0–2.1	
> 50.000	388	60.5	1.4	1.1–1.6		82	12.5	1.7	1.1–2.4	

(*) sometimes or often vs never

(°) current vs never or in the past only

+ ORs are adjusted for all listed variables

## Discussion

This paper reports the findings of a large population-based survey on the knowledge, recommendation and practice of CAM among GPs in Tuscany. Unlike previous studies, the random sampling strategy and the high response rate ensure that the sample is representative and that the results can be generalised. Tuscany is one central region of the country where the populations' use of CAM, which in Italy decreases gradually from the North to the South of the country [18], is similar to the national average [9]. Should the same geographical gradient apply to physicians, our findings may be considered indicative of GPs' level of knowledge and behaviours regarding CAM throughout the entire country.

In the present study over half of the respondents reported to recommend CAM to their patients while a far smaller fraction (13%) acted as CAM practitioners themselves. Published figures on the practice of CAM by primary care physicians vary greatly across countries, ranging from 8% [19] and 13% in Israel [20]; 16% [21] and 20% in the UK [22,23]; 16% in Canada [24]; 20% [25] and 38% in Australia [26]; 30% in New Zealand [27]; 47% in the Netherlands [28], and up to 95% in Germany [29]. The popularity of different types of CAM also appear to vary geographically, with homeopathy being practised by 40% of Dutch GPs [28] and herbal medicine being favoured in Germany [29]. Year of data collection, different methodological choices and sampling strategies can certainly explain part of the variation [16]. The frequency of CAM provision by GPs may also be related to the extent to which it is demanded by patients and also to different policies of financial coverage across countries.

To our knowledge, the relationship between personal life-styles and attitudes towards CAM among physicians has not been previously explored. In this study factors such as following a vegetarian or macrobiotic diet and practising physical activity appear to be associated with the professional use of CAM. These findings support the theory that a holistic view of health and health care can be one of the possible determinants of GPs' interest in this type of medicine [5,30].

The conditions for which physicians apply CAM are similar to those for which consumers themselves use these therapies, the most common being pain syndromes, psychological conditions, and chronic illnesses in general. Indeed these conditions, often dealt with in primary care, may still lack fully satisfactory treatments through conventional medicine. This "effectiveness gap" may partially explain the appeal of CAM to primary care physicians [31]. Yet, lack of conventional effective treatment cannot in itself justify the adoption of another treatment for which evidence of effectiveness is lacking and for which there is no control over the training and experience of the practitioners applying it. Under this aspect, our study has shown some worrisome findings. Among almost two hundred Tuscan family doctors currently practising CAM, more than one third reported that no specific certificated training was completed or in progress. This was the case for approximately 40% of GPs practising acupuncture and homeopathy and 82% GPs practising phytotherapy. Admittedly, the absolute number of CAM practitioners without specific training is small; yet the issue of professional competence in CAM is an important concern that has been highlighted also by other authors [4,7,32]. In Italy CAM training is mostly offered by private schools or associations and there is little control over the quality of the education provided. Even in countries where CAM is more widely included in medical curricula, education and certification of CAM practitioners is still an existing problem [12,32]. The rapidly spreading public enthusiasm for CAM, coupled with the increasing acceptance of a consumerist and market-driven approach to health care, may push some physicians to respond to the patients' requests without having appropriate training. In addition, the perception of CAM as generally safe in terms of side effects, although incorrect [33], may contribute to its uncontrolled use.

Nevertheless, our study reveals that as many as 42% of Tuscan GPs never recommend CAM, principally for the insufficient evidence of its effectiveness. The lack of proper randomized controlled trials in the field of CAM and of substantial evidence of its effectiveness is a serious concern [12,13,27,34].

## Conclusion

CAM knowledge among GPs is not as widespread as the public demand seems to require, and the scarce evidence of CAM effectiveness hinders its professional use among a considerable number of GPs. Some CAM practitioners maintain that the assumptions underlying CAM treatment and procedures make it unsuitable for conventional scientific assessment. Yet, many of the methodological problems encountered are common to other therapeutic research [35]. Methodologically sound CAM research is essential to guide physicians' behaviour, to safeguard patients' safety, and to assist policy-makers in developing regulations for usage.

## Competing interests

The author(s) declare that they have no competing interests.

## Authors' contributions

MG participated in the design of the study and its coordination, participated in the draft of the questionnaire and data analysis, and carried out the data collection and draft of the manuscript.

MC participated in the design of the study and its coordination, conceived and drafted the questionnaire, and helped to draft the manuscript. MDF performed the data analysis and helped to revise the manuscript. EB participated in the design of the study and its coordination, and helped to revise the manuscript. All authors read and approved the final manuscript.

## Pre-publication history

The pre-publication history for this paper can be accessed here:


